# Exploring the Relationship Between Continuously Monitored Vital Signs, Clinical Deterioration, and Clinical Actions

**DOI:** 10.3390/jcm14010281

**Published:** 2025-01-06

**Authors:** Roel V. Peelen, Yassin Eddahchouri, Ilse M. Spenkelink, Harry van Goor, Sebastian J. H. Bredie

**Affiliations:** 1 Department of Internal Medicine, Radboud University Medical Center, 6525GA Nijmegen, The Netherlands; bas.bredie@radboudumc.nl; 2 Department of Surgery, Radboud University Medical Center, 6525GA Nijmegen, The Netherlandsharry.vangoor@radboudumc.nl (H.v.G.); 3 Health Innovation Lab, Radboud University Medical Center, 6525GA Nijmegen, The Netherlands

**Keywords:** general ward, continuous vital signs monitoring, patient safety, clinical deterioration, predictive monitoring

## Abstract

Continuous monitoring on the general ward leads to more and earlier interventions to prevent clinical deterioration. These clinical actions influence outcomes and may serve as an indicator of impending deterioration. This study aims to correlate clinical actions with clinical endpoints and deviating vital signs. **Methods:** This cohort study prospectively charted all patients undergoing continuous vital sign monitoring on a gastro-intestinal and oncological surgery, and an internal ward of an academic hospital in The Netherlands from 1 August 2018 till 31 July 2019 (METC 2018-4330, NCT04189653). Clinical actions recorded in electronic medical records were analyzed to assess correlations with patient outcomes, hospital length of stay, and alarming monitoring minutes. **Results:** A total of 1529 patients were included, of which 68 patients had a negative clinical endpoint. There were 2749 clinical actions recorded. Clinical actions correlated to negative clinical endpoints (ρ = 0.259; *p* < 0.001, OR: 3.4 to 79.5) and to the length of stay (ρ = 0.560; *p* < 0.001). Vital sign deviations correlated with clinical actions (ρ = 0.025–0.056; *p* < 0.001–*p* = 0.018). In the last 72 h before a clinical endpoint, for alarming minutes, this correlation with clinical actions was more pronounced (ρ = 0.340, *p* < 0.001). **Conclusions:** Predefined clinical actions performed on admitted general ward patients correlated with negative endpoints, an increased length of stay, and with deviating vital signs, especially in the period directly preceding severe deterioration. Clinical actions have potential as an intermediate measurement of deterioration.

## 1. Introduction

Unnoticed clinical deterioration remains a worrisome problem in general wards. It contributes to negative clinical endpoints such as morbidity, prolonged hospital admissions, ICU transfer, and mortality [1,2]. Research uses clinical endpoints as outcomes but also as targets for detection [3]. With the introduction of continuous monitoring, awareness of impending deterioration can trigger timely interventions that may prevent negative endpoints [4,5,6]. However, this creates a paradox: effective prediction of endpoints may be counteracted by interventions initiated based on those predictions. Predicting alerts for initiating interventions may, therefore, be more meaningful than predicting clinical endpoints that are subsequently averted. In a perfect scenario, this could lead to a self-regulating system where interventions based on deviating vitals would normalize these vitals and prevent further clinical deterioration.

**Figure 1:**
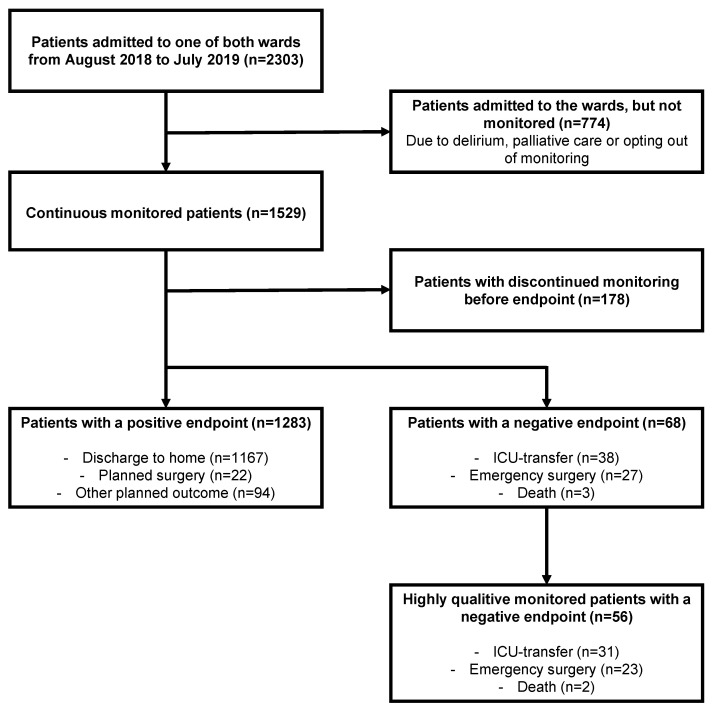
Population flow diagram.

Continuous monitoring, however, can result in a surplus of alarms, of which the clinical significance is not yet fully understood. Our previous research showed a 7-fold increase in false alarms with continuous monitoring compared to periodic monitoring, with other studies reporting even higher figures [1,7]. Patients who experienced negative clinical endpoints (RRT activation, ICU transfer, emergency surgery, or death) had more alarming minutes of vital signs throughout their admission [7]. Therefore, alarming minutes might be early indicators of impending deterioration. In patients without negative endpoints, a similar accumulation of alarming minutes might have prompted timely interventions that prevented further deterioration. These patients might have benefited from continuous monitoring, as demonstrated by the reduced escalations found in previous research [5].

To investigate the aforementioned paradox and provide insight into the numerous alarms from continuous monitoring, focusing on clinical actions as a new outcome could offer a solution. Predictions of classical endpoints over multiple days are often less useful for clinicians, who need to make timely interventions to prevent deterioration. They rely on actionable triggers from continuous monitoring trends for early detection. Thus, examining the relationship between clinical actions and traditional endpoints is relevant. If such a relationship exists, predicting clinical actions could be highly valuable. Using clinical actions as a precursor helps understand deterioration by identifying actionable triggers from monitoring data that correlate with interventions preventing adverse outcomes [8,9,10,11]. This study aims to determine the relationships between clinical actions and clinical deterioration. To this end, we test two hypotheses: (1) the number of clinical actions correlates with negative clinical endpoints and admission duration, and (2) clinical actions correlate with the number of alarming minutes during vital sign monitoring preceding clinical deterioration [8,9,10]. By testing these relationships, we want to investigate the potential of vital sign monitoring in directing clinical actions to prevent clinical deterioration.

## 2. Materials and Methods

This study was performed at the gastro-intestinal oncological surgery ward and internal medicine ward of the Radboud University Medical Center in the Netherlands. All patients were connected to a continuous vital sign monitor as a standard of care. During admission, real-time vital signs were available for caregivers. All patients admitted during one year (August 2018 to July 2019) who were connected within 12 h of admission were included. Patients were excluded from analysis if monitoring was stopped due to the patient’s objection or inability to undergo monitoring, such as for patients with hyperactive delirium, contact allergies, or initiation of non-resuscitation status. For further analysis, only the data of patients with a complete follow-up until the endpoint were used (Figure 1).

This study was performed according to the declaration of Helsinki on medical research and was approved by the Medical Ethics Committee (METC 2018-4330) and registered at ClinicalTrails.gov (NCT04189653). The need for signed informed consent was waived due to the standard of care nature of the intervention, opting out of monitoring, or inclusion in our study database. This study was conducted according to the Strobe method and checklist (https://www.equator-network.org/reporting-guidelines/strobe/, accessed on 21 August 2024).

### 2.1. Clinical Data

The clinical condition of all included patients was reviewed daily. To this end, daily readouts of the electronic medical records (EMRs) with all interventions and registrations per patient were reviewed, as well as all medical and nursing notes. This was within 24 h to prevent selections bias based on outcomes and to document these registrations in as ‘prospective’ a manner as possible. All clinical interventions, observations, and diagnoses were registered. Any anomalies in treatment related to clinical deterioration were time-stamped to the nearest minute using the most relevant and reproducible datapoints, for instance the moment of scanning the medication for verification. For each new type of intervention, diagnostic or another action, a reproducible and accurate datapoint was identified and used for annotation in the database. Also, the endpoints were registered according to these rules. New symptoms were registered if they were unexpected and were added to the clinical problem list. This was performed by a physician (RP or YE). If multiple interventions were registered around the same time, all were counted. Clinical endpoints were categorized into discharge, transfer to another ward, transfer to an ICU ward, unplanned surgery, and death. When monitoring was discontinued for longer than 24 h, follow-up was stopped, and the moment of discontinuation was registered. All registrations were entered in customized digital forms of a validated database for clinical research (Castor EDC, Castor EDC BV, Amsterdam, The Netherlands).

### 2.2. Clinical Actions as an Outcome

After the data-gathering period, the registrations were checked for improbabilities and duplicate registrations. All records were reviewed and discussed, to obtain consensus on the registered interventions, the observations, and the diagnoses that were potentially deterioration-related actions. All actions had to be traceable to specific orders in the EMR to enhance reproducibility. Actions used in this study had to deviate from the regular course and be causally linked to potential deterioration, based on expert opinion. Additionally, actions should be suitable to be used as an intermediate outcome, e.g., actions that are meant to prevent new or further deterioration. The final set of clinical actions consisted of newly recorded symptoms and diagnoses, unplanned medication changes, physical interventions, and consultations. The list of used actions can be found in Appendix A Table A1. The occurrences of any of these selected actions are referred to as ‘clinical actions’, which are considered as an intermediate outcome measurement of deterioration.

### 2.3. Vital Sign Monitoring Data and Alarming Minutes

As part of standard care, all patients were connected to the ViSi Mobile (Sotera Wireless, San Diego, CA, USA) device. Therefore, heart rate, respiration rate, blood oxygen saturation, and cuffless blood pressure were continuously measured with a complete set of these vitals registered to our database every minute. For evaluation, we used these one-minute data and a corresponding risk score, the Visensia Safety Index (VSI), which is an automated algorithm that scores the vital signs based on their combined deviation from normality (OBS Medical, Oxford, UK). The VSI can include core temperature as a parameter. The ViSi Mobile device measures skin temperature, not a core temperature. Since this skin temperature is significantly subjected to external influences and, therefore, not widely used, we have excluded temperature from the analysis. Alarming minutes were defined by a VSI of 3 or higher as suggested and tested by the supplier and counted per unique minute. When values of vital parameters were missing within a minute, these (seconds) were left blank. Outlier values that could not be associated with any physiological circumstance were filtered out and discarded for analysis.

### 2.4. Defining Endpoints, Periods, and Analysis

We analyzed the correlation of the accumulated number of clinical actions during admission per patient with conventional endpoint outcomes. First, we tested if the number of clinical actions was correlated to negative endpoints (ICU-transfer, emergency surgery, or death) using Spearman’s rank correlation coefficients. Subsequently, we calculated the Relative Risk for escalation of care within categories with an increasing number of actions. In admissions without a negative clinical endpoint, we tested for a correlation between the number of actions and the length of stay and corrected for an extended duration of the admission using Spearman’s rank correlation coefficients and Mann–Whitney U tests after positive Kruskal–Wallis tests.

As a second step, we investigated the correlation between the occurrence of clinical actions and continuously monitored vital signs. For this purpose, we divided the recordings into arbitrary 8 h intervals. This was based on the duration of the nurse shift and the maximum interval between measurements according to the Early Warning Score protocol applied in our hospital. Next, we determined the average of the different vital parameters and assessed their average deviation from normality by means of the VSI, the number of alarming minutes defined by a VSI above 3, and the number of actions in the 8 h interval. Then, we tested the correlation between alarming minutes and the occurrence of actions in the first 120 h for all admissions. The 120 h period was considered as the median admission time of the study population. Additionally, we explored the correlation between the occurrence of actions and the number of alarming VSI minutes in the last 72 h before a negative endpoint. Therefore, we selected admissions which ended in a negative endpoint and that had at least 50% of data capture in the last 72 h (Figure 1). Considering the smaller population, we lengthened the intervals to 12 h to gather enough clinical data in each of these periods. The duration of 72 h was chosen arbitrarily as a maximum period for clinical useful predictions and a duration that is in proportion to the average length of admission.

For statistical analyses we used IBM SPSS 25 (SPSS, Inc., Chicago, IL, USA). Descriptive statistics are presented as the mean and standard deviation (SD) or median and interquartile range (IQR), depending on the skewness of the data distribution, as assessed with the Shapiro–Wilk test. For confidence intervals of proportions, we used the standard error to calculate a 95% confidence interval. Spearman’s rank correlation coefficients (Spearman’s ρ) were used as a nonparametric measure of correlations. Mann–Whitney U tests and Kruskal–Wallis tests were used to compare medians and the distribution of non-normally distributed data. A *p*-value less than 0.05 was considered statistically significant. Correlations with a coefficient below 0.3 were classified as weak, correlations between 0.3 and 0.5 were deemed moderate, and coefficients above 0.5 were categorized as strong.

## 3. Results

### 3.1. Patient and Data Characteristics

A total of 1529 patients were continuously monitored and were eligible for overall analysis, of which 68 patients had a negative clinical outcome, namely ICU-transfer, emergency surgery, or death. This results in a rate of 4.4%, which is comparable to our 3-year average general ward hospital rate of 4.2% [2]. Of these 68 patients, 56 patients were eligible for more selective analysis of the last 72 h, having ‘highly qualitative’ monitoring with at least 50% of data capture in that last period (Figure 1). Table 1 shows the characteristics of the included patients. A total of 10,074,490 min of continuous vital sign monitoring over a total of 8890 patient days were recorded, of which 51,842 min were alarming minutes based on a Visensia Safety Index (VSI) score of 3 or higher. This amounts to 0.5% of the measured minutes being alarming. Of the 10,396 registered actions, 3175 clinical actions were selected on the basis of predefined criteria. Of these, 2749 actions were registered in the population with a successful follow-up, with 642 actions in the population with a negative outcome and 566 actions in the highly qualitatively monitored patients with a negative endpoint (Appendix A Table A1).

### 3.2. Step 1: Correlation of Clinical Actions and Outcomes

There was a significant positive correlation between the number of clinical actions and negative endpoints in the overall population (ρ = 0.259; *p* < 0.001). This is illustrated in Table 2 by a significantly higher Relative Risk ratio for negative clinical endpoints between almost all groups with an increasing number of clinical actions. Relative Risk ratios ranged from 2.9 for the group with 6 to 10 actions compared to 1 to 5 actions, to a risk ratio of 42.3 (highest) when comparing the group with more than 15 actions to the group with no actions.

When investigating patients without negative clinical endpoints, a longer admission duration was found in patients with more clinical actions (Spearman ρ = 0.560; *p* < 0.001). A moderate correlation between the number of actions per hour and the length of stay was observed (Spearman ρ = 0.394; *p* < 0.001). When testing the groups with an increasing number of actions against the previous group, we also found significant differences (Table 3).

### 3.3. Step 2: Correlation of Clinical Actions and Vital Signs

We found significant but weak correlations between the number of clinical actions and the 8 h averaged heart rate, respiration rate, and blood pressure in the first 120 h of all admissions, as shown in Table 4. The correlation also did not increase when using the VSI as a measure for vital sign deviation from normality instead of the vital parameters themselves. Furthermore, the correlation with the number of alarming VSI minutes was not significant.

**Figure 2:**
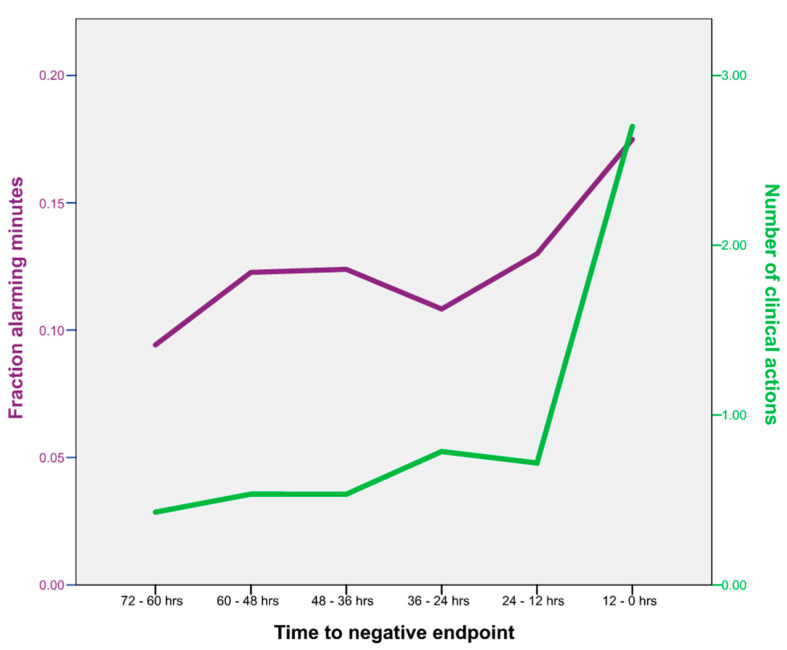
Graphical representation of the trend of alarming VSI minutes and the number of clinical actions.

When examining the relation between the number of clinical actions and the alarming minutes of the VSI in the 72 h prior to a negative clinical endpoint outcome, a moderate correlation was observed (ρ = 0.340, *p* < 0.001). A break in the trend is seen in the last 12 h before the endpoint when the number of clinical actions rises more sharply than the fraction of alarming VSI minutes (Figure 2).

## 4. Discussion

This study explored clinical actions during hospital admission as an intermediate outcome measurement in the run-up to clinical deterioration and the relation of clinical actions to continuously monitored vital signs. Results confirmed both hypotheses: (1) that the frequency of clinical actions correlated with negative clinical endpoints and prolonged admission duration, and (2) the results demonstrated a correlation between the number of clinical actions and alarming minutes with continuous monitoring, preceding severe clinical deterioration.

We previously have reported that the implementation of continuous monitoring reduces negative clinical endpoints and identifies early signs of deterioration through increased alarming minutes in patients requiring care escalation [5,7]. We expected that these increased alarms might reflect early signs of deterioration which would initiate clinical actions by care providers to prevent further deterioration. Now, we show that patients who undergo more clinical actions have an increased Relative Risk of a negative clinical endpoint. The frequency of clinical actions also correlated with a longer hospital stay which may suggest more complex and challenging clinical situations. For patients with severe illness, single interventions are often insufficient, and multiple, progressively escalating therapeutic adjustments are needed. When these actions are effective, vital signs stabilize with subsequent improvement of the clinical situation. The number of interventions required for normalization may thus indicate the severity of deterioration even without reaching common negative clinical endpoints. However, it is essential to note that these correlations do not imply causality. Clinical actions are responses to deviations in vital signs and clinical cues, representing standard efforts by care providers to prevent further deterioration.

Unlike traditional endpoints, which often represent binary outcomes such as deterioration or no deterioration, clinical actions as an outcome provide a more nuanced and gradual scale. This allows for the evaluation of subtle physiological changes and the effectiveness of interventions at earlier stages of clinical deterioration. Continuous vital sign monitoring and advanced scoring systems enable the identification of early signs of bodily dysregulation before serious clinical deterioration occurs. By focusing on clinical actions as intermediate outcomes, future research can explore how vital sign markers and scores relate to early symptoms of dysregulation, offering opportunities to intervene earlier and more effectively. Such an approach aligns with the shift in clinical practice from reacting to late-stage deterioration to proactively managing early signs of instability [4]. In this context, a clinical action does not only serve as a marker of the severity and complexity of a patient’s condition but also as a validation of the outcomes of continuous monitoring technology and associated algorithms. By leveraging these intermediate outcomes, researchers can refine predictive models and scoring systems by ensuring sensitivity in the detection of early changes while minimizing false alarms.

In patients experiencing a negative endpoint, alarming minutes significantly correlated with clinical actions, supporting our hypothesis that such clinical actions reflect intermediate stages of deterioration detected by vital sign deviations. However, the alarming minutes do not correlate with clinical actions in the overall population. We assume that the deviation in the vital signs is not reaching the relative high alarm threshold of the algorithm. Since individual vital parameters correlated weakly with clinical actions (ρ = 0.025 to 0.056), the strength of these correlations is insufficient for clinical use. Factors such as circadian rhythm and activity levels likely influenced vital signs, outweighing the correlation with clinical actions. Although we did not take these effects into account within our study, other research has shown that continuous monitoring is sensitive to circadian rhythm and increased activity [12,13,14,15], which also confers diagnostic potential. The recent literature suggests that circadian rhythm, especially the absence of it, is a potential indicator for clinical deterioration [12,13,14,15,16,17]. Various research has shown a decrease and subsequent recovery of circadian rhythm after surgery or during critical illness. Also, van Ede showed that an increase in activity, which is a sign of recovery, led to an increase in false alarms of vital signs during this activity [13]. This can even be part of the recovery plan, as physiotherapy frequently uses temporary deviations in vital signs as training goals [18].

In this study, we have explored the concurrent occurrence of vital sign deviations defined by alarming minutes and clinical actions and showed meaningful correlations. However, this relationship could also be examined from a different perspective. Our current research approach focuses on deterioration as reflected by abnormal vital signs and interventions aimed at reversing this process. A different approach is to study clinical actions in normalizing vital sign parameters. Such an approach might address questions like whether the intervention was sufficiently effective and whether it led to restoration towards normal vital signs. Distinguishing effective and non-effective interactions based on bodily dysregulation may refine the definition of appropriate clinical care. Although this perspective lies beyond the scope of the current study, it represents a promising avenue for further research.

The low complication rate in our general ward cohort reflects real-world conditions that are more realistic than those of other research [1,10,12]. Our complication rate of 4.4% is comparable to or slightly lower than incidences between 5 and 8% in other studies [1,19,20]. In the context of a low complication rate, the relative influence of confounding factors may have become more pronounced. This assumption aligns with the nature of diagnostic and screening test results. When the baseline prevalence of complications is low, the positive predictive value decreases, and the subtle associations between predictive factors and outcomes are more easily masked by noise from confounders [21]. Even when clinical actions are used as a more frequently measured intermediate outcome, the low incidence of adverse events limits the model’s sensitivity and contributes to weaker correlations within the dataset. Sub-analysis in patients requiring care escalation revealed stronger correlations between alarming minutes and clinical actions, underscoring the potential of continuous monitoring in high-risk, e.g., high-incidence, subgroups.

Continuous vital sign monitoring is increasingly replacing intermittent measurements for detecting clinical deterioration [1,4,8]. While strict thresholds are appropriate for spot measurements, other threshold and alarming strategies could enhance the detection of deterioration when using continuous monitoring [4,12]. Therefore, it is important that we learn to differentiate changes in vital signs indicative of deterioration from changes caused by other factors. Clinical actions, used as an intermediate outcome, offer a promising framework to link vital sign deviations with deterioration. This approach can improve the understanding of how vital sign deviations drive interventions, enabling earlier detection of at-risk patients and timely countermeasures. Incorporating factors like activity and circadian rhythm into predictive models may further enhance accuracy by filtering non-pathological fluctuations, supporting better care planning, resource optimization, and even early discharge decisions [22].

Advances in machine learning and artificial intelligence highlight the need to define clinical deterioration gradients precisely [3]. Our study’s novel use of intermediate outcomes preceding conventional endpoints provides a framework for earlier detection and intervention. We experimentally expanded the definition of clinical deterioration, which could help refine predictive algorithms and improve patient outcomes.

### Strengths and Limitations

This study’s strength lies in its real-world population with minimal patient selection, enhancing generalizability. Continuous vital sign monitoring as standard care allowed healthcare professionals to follow up on alarms integrated in their care. Although we included a surgical and internal medicine ward, we believe that the diversity in patients with and without an operation reflects the population of an average hospital. For further research considering other specialty wards, the predefined actions in this study should be expanded with relevant specialty-specific interventions. Nevertheless, we believe that most deteriorations show many of the same causes and similar courses for the different specialties, for example infections, shock, ischemia, and metabolic disorders.

Despite careful registration, rare discrepancies between the registered time and actual time of clinical actions may have occurred. This, however, is unlikely to have significantly impacted our findings. We were not able to correct nor test for confounding factors affecting vital sign parameters, such as circadian rhythm. This may have hindered the identification of patients who might have benefitted from an intervention to prevent deterioration.

With our study being in a standard care clinical setting, the quality of monitoring data was not only affected by the clinical workflow but also by technical difficulties with the monitoring devices, logistic challenges, working agreements (e.g., hygiene and infection prevention protocol), and external factors. This led to the elimination of a relatively small proportion of patients for further analysis due to limited data.

## 5. Conclusions

This study conducted on two general wards, a surgical and an internal medicine ward, suggests that clinical actions serve as an intermediate outcome, revealing a correlation between clinical actions, negative clinical endpoints, and length of stay. Additionally, in patients who experience severe clinical deterioration, alarming minutes correlated with concurrent clinical actions. Further research is needed to explore the potential of continuous vital sign monitoring and scoring to identify the appropriate and needed clinical intervention and to monitor the effect of the intervention, all intended to prevent clinical deterioration.

## Figures and Tables

**Table 1:** Patient characteristics.

	Overall Monitored Population	Highly Qualitatively Monitored Patients with a Negative Clinical Endpoint
No. of patients	1529	56
Medical/Surgical admissions	1005/524	24/32
Age (years)	60.1 ± 25.1	63.0 ± 23.7
Male sex (%)	51.9%	66.1%
ASA (I/II/III/IV)	48/695/793/23	0/19/36/1
Full code on admission	87.0%	85.7%
Endpoints		
Discharge or planned outcome	1283	-
Monitoring stopped	178	-
ICU-transfer	38	31
Unplanned Surgery	27	23
Death	3	2
Length of Stay (IQR)	100.7 (53.0–176.0)	98.0 (65.8–165.1)

**Table 2:** Relative Risk ratios for negative endpoints when comparing groups with increasing number of clinical actions.

	1–5 Actions (*n* = 496)	6–10 Actions (*n* = 91)	11–15 Actions (*n* = 34)	>15 Actions (*n* = 15)
0 actions (*n* = 715)	5.9 (2.7–12.8; *p* < 0.001)	17.3 (7.7–38.7; *p* < 0.001)	21.7 (8.9–53.1; *p* < 0.001)	42.3 (18.3–97.7; *p* < 0.001)
1–5 actions (*n* = 496)	-	2.9 (1.7–4.9; *p* = 0.001)	3.6 (1.9–7.0; *p* = 0.001)	7.1 (4.0–12.7 *p* < 0.001)
6–10 actions (*n* = 91)	-	-	1.3 (0.6–2.5; *p* = 0.524)	3.8 (1.3–4.5; *p* = 0.005)
11–15 actions (*n* = 34)	-	-	-	1.9 (0.9–4.0; *p* = 0.077)

**Table 3:** Correlation between clinical actions and length of stay of discharged patients, between groups with increasing number of clinical actions.

	Length of Stay		Actions/h	
	Median [IQR] (h)	MWU to Previous Group	Median [IQR] (Actions/h)	MWU to Previous Group
0 actions (*n* = 707)	73.0 [43.0–89.9]	-	0	-
1–5 actions (*n* = 463)	144.0 [91.0–215.0]	U = 81,742.5 Z = −14.5 *p* < 0.001	0.014 [0.009–0.023]	*p* < 0.001
6–10 actions (*n* = 75)	215.0 [168.0–333.0]	U = 9444.0 Z = −6.3 *p* < 0.001	0.033 [0.022–0.043]	U = 5981.0 Z = −9.1 *p* < 0.001
11–15 actions (*n* = 28)	368.0 [220.0–608.5]	U = 588.5 Z = −3.4 *p* = 0.001	0.036 [0.021–0.059]	U = 992.0 Z = −0.4 *p* = 0.667
>15 actions (*n* = 10)	494.5 [348.8–856.0]	U = 100 Z = −1.326 *p* = 0.194	0.044 [0.021–0.063]	U = 120.0 Z = −0.7 *p* = 0.524

MWU = Mann–Whitney U test.

**Table 4:** Correlations between clinical actions and vital signs in first 120 h of admission of the overall monitored population.

	Spearman’s Rho	
Heart rate	0.056	*p* < 0.001 *
Respiration rate	0.032	*p* < 0.001 *
Blood oxygen saturation	0.003	*p* = 0.718
Systolic blood pressure	0.021	*p* = 0.018 *
Mean arterial blood pressure	0.025	*p* = 0.005 *
Visensia Safety Index		
Averaged VSI	0.029	*p* < 0.001 *
Alarming VSI minutes	0.007	*p* = 0.327

* = significant correlation.

## Data Availability

Due to the confidential nature of the data and the potential for individual identification, the data will not be made publicly available online. However, the data can be made accessible for review in consultation with the corresponding author.

## Appendix A

**Table A1:** List of all registered actions in the clinical trajectory of the overall population.

	Number of Occurrences
Total	2749
REGISTERED SYMPTOMS	485
Abnormal heart rhythm	28
Abnormal blood pressure reading	2
Abnormality of breathing	110
Hypoxia	15
Abnormalities of bowl moments	12
Abnormalities of urination	64
Somnolence/coma	11
Bacteriemia	6
Fever	201
Extreme abnormalities of blood chemistry	20
Extreme abnormalities of kidney function	1
Extreme abnormalities of liver function	5
Abnormality of blood glucose	10
DIAGNOSTICS	693
Imaging	378
X-thorax	144
CT-scan	162
Ultrasound	43
Other imaging	29
Invasive diagnostics	58
Lumbar punction	16
Colonoscopy, endoscopy of gastroscopy	18
Cardiac angiography	4
Bronchoscopy	5
Biopsy	15
Electrocardiogram	49
Pathology and microbiology	136
Urgent laboratory inquiries	65
Other diagnostics	7
MEDICATIONS: STARTED OR CHANGED	1152
Analgesia	197
Antiarrhythmic	32
Antibiotics	297
Antihypertensive	32
Antithrombotic	10
Diuretics	134
Oxygen therapy or respiratory medications	59
Blood products	37
Infusion fluids	138
Glucose	39
Electrolytes	18
Steroids	32
Others	127
PHYSICAL INTERVENTIONS	240
Rapid Response Team activation	59
Punction or drainage	81
Small surgical intervention	20
Endoscopic probe placement	39
Bronchial toilet	6
Advanced urinary interventions	9
Central venous catheter placement	18
Other advanced medical interventions	8
CONSULTATIONS	179
Anesthesiology, pain, and palliative care	13
Cardiology	43
Surgery	11
Neurology	13
Pulmonology	25
Internal medicine	22
Urology	16
Gastroenterology	11
Other	25
